# Transcriptome differences in the hypopharyngeal gland between Western Honeybees (*Apis mellifera*) and Eastern Honeybees (*Apis cerana*)

**DOI:** 10.1186/1471-2164-15-744

**Published:** 2014-08-30

**Authors:** Hao Liu, Zi-Long Wang, Liu-Qing Tian, Qiu-Hong Qin, Xiao-Bo Wu, Wei-Yu Yan, Zhi-Jiang Zeng

**Affiliations:** Honeybee Research Institute, Jiangxi Agricultural University, Nanchang, Jiangxi 330045 China

**Keywords:** *Apis mellifera*, *Apis cerana*, Hypopharyngeal gland, Digital gene expression, Differentially expressed gene

## Abstract

**Background:**

*Apis mellifera* and *Apis cerana* are two sibling species of Apidae. *Apis cerana* is adept at collecting sporadic nectar in mountain and forest region and exhibits stiffer hardiness and acarid resistance as a result of natural selection, whereas *Apis mellifera* has the advantage of producing royal jelly. To identify differentially expressed genes (DEGs) that affect the development of hypopharyngeal gland (HG) and/or the secretion of royal jelly between these two honeybee species, we performed a digital gene expression (DGE) analysis of the HGs of these two species at three developmental stages (newly emerged worker, nurse and forager).

**Results:**

Twelve DGE-tag libraries were constructed and sequenced using the total RNA extracted from the HGs of newly emerged workers, nurses, and foragers of *Apis mellifera* and *Apis cerana*. Finally, a total of 1482 genes in *Apis mellifera* and 1313 in *Apis cerana* were found to exhibit an expression difference among the three developmental stages. A total of 1417 DEGs were identified between these two species. Of these, 623, 1072, and 462 genes showed an expression difference at the newly emerged worker, nurse, and forager stages, respectively. The nurse stage exhibited the highest number of DEGs between these two species and most of these were found to be up-regulated in *Apis mellifera*. These results suggest that the higher yield of royal jelly in *Apis mellifera* may be due to the higher expression level of these DEGs.

**Conclusions:**

In this study, we investigated the DEGs between the HGs of two sibling honeybee species (*Apis mellifera* and *Apis cerana*). Our results indicated that the gene expression difference was associated with the difference in the royal jelly yield between these two species. These results provide an important clue for clarifying the mechanisms underlying hypopharyngeal gland development and the production of royal jelly.

**Electronic supplementary material:**

The online version of this article (doi:10.1186/1471-2164-15-744) contains supplementary material, which is available to authorized users.

## Background

The hypopharyngeal gland (HG), which is a pair of glands located in the head of worker bees, is composed of clusters of acini, which deliver secretions (royal jelly) into a collecting duct that runs to the mouthparts. The main function of the HG is to produce and secrete the protein components of royal jelly, which is fed to the queen and larvae. The secretory activity and function of HGs are age-dependent [1]. In newly emerged workers, the HGs are small and not fully developed. After that, the secretory activity of HGs could reach a peak within 6–12 days, and their main function at this stage is to synthesize and secrete royal jelly to feed larvae. The HGs then gradually degrade during the forager stage, and their protein secretion changes to the secretion of enzymes for brewing honey [2–4]. In addition, the HG has been reported to display flexible secretory activity in response to the needs of the feeding brood [5].

During the transition from newly emerged workers to foragers, the HGs show a marked change not only in size but also in protein synthesis. Some proteins, including alpha-glucosidase [2, 6], amylase and glucose oxidase [3], have been reported to display an age-dependent expression pattern in the HGs of workers. Ohashi identified a 64-kDa protein (RJP57-1) that is expressed specifically in the HGs of nurse bees and a 56-kDa protein that is expressed in the HGs of nurse bees and forager bees [4]. Santos *et al.* identified the protein complement of the HGs of Africanized nurse bees (*Apis mellifera L.*) and found that almost all of them were related to the MRJP family and associated with the metabolism of carbohydrates and energy [7]. Using proteomics method, Feng *et al.* analyzed the protein profile of six developmental stages of the *Apis mellifera* HGs and identified many proteins, including MRJPs and proteins involved in cytoskeleton, antioxidant activity, developmental regulation, and carbohydrate, lipid and protein metabolism [8]. Moreover, Li *et al.* analyzed the protein expression difference in hypopharyngeal gland development between Italian and royal jelly-producing workers (*Apis mellifera L.*) through proteomics [9]. Their results demonstrated that a high royal jelly-producing honeybee strain significantly up-regulates a large group of proteins involved in metabolism of carbohydrates, nucleotides, amino acids, and fatty acids, proteins involved in protein biosynthesis, energy production, development, antioxidation, and protein folding, and transporter and cytoskeleton proteins. Recently, Liu *et al.* analyzed the gene expression difference between five developmental time points of HGs in *Apis mellifera* and identified many DEGs [10].

*Apis mellifera* and *Apis cerana*, as representative honeybee species of the East and West, are two important honeybee species that are widely bred and studied. Recent studies on these two species have revealed that both geographical isolation and long-term evolutionary divergence are responsible for their differences in key biological characteristics, such as shape, individual development, and living habit [11]. *Apis cerana* is adept at collecting sporadic nectar in the mountain or forest region and exhibits stiffer hardiness and acarid resistance as a result of natural selection. *Apis mellifera* has the advantage of yielding royal jelly which is one of the main differences between these two honeybee species [11]. A previous study indicated that the mean length and the acini number of the *Apis mellifera* HGs were significantly greater than those of the *Apis cerana* HGs, and the royal jelly yielding ability of *Apis mellifera* was more than ten-fold higher than that of *Apis cerana* [12]. Fang *et al.* compared the protein profiles of royal jelly produced by *Apis mellifera ligustica* and *Apis cerana cerana* using proteomic approaches and identified that royal jelly proteins (MRJPs), peroxiredoxin 2540, and glutathione S-transferase S1 were differentially expressed [13]. However, no studies on the transcript and/or protein differences between the HGs of *Apis mellifera* and *Apis cerana* have been reported. Detecting the gene expression difference in HGs between these two sibling species is important for understanding the mechanism of high royal jelly production.

The completion of the honeybee (*Apis mellifera L.*) genome sequencing [14] and the development of high-throughput sequencing methods provide the possibility for us to investigate the genome-wide gene expression profile. The aim of this study was to use DGE-tag analysis to identify genes specifically expressed in HGs that were associated with a significant difference in the production of royal jelly between *Apis mellifera* and *Apis cerana*. Through DGE sequencing and rigorous screening, we identified 1417 DEGs between *Apis mellifera* and *Apis cerana*. Our study provides valuable data for clarifying the molecular mechanism of HG development and a high yield of royal jelly in honeybees.

## Results and discussion

### DGE library sequencing

Twelve DGE-tag libraries were constructed and sequenced using the total RNA extracted from the HGs of *Apis mellifera* and *Apis cerana* at the three developmental stages (newly emerged worker, nurse, and forager), which are three typical developmental stages of HGs. For each library, HGs dissected from 60 workers were pooled as a sample to construct the library. The sequencing results showed that the two biological replicates of each sample have a high reproducibility (0.88 < R < 0.99) (Additional file 1: Figure S1), suggesting the high reliability of the sequencing results. After the low-quality tags, tags with a copy number less than two, and adaptor sequences were filtered, the remaining clean tags of each library were approximated 5.8 million, and the percentage of clean tags among the raw tags in each library was approximately 98% with the exception of *Aml*_forager 1 and *Aml*_forager 2, which were approximately 62.65% (Table 1 and Additional file 2: Figure S2). The percentages of unambiguous tags that could be mapped to reference genes (ftp://ftp.ncbi.nih.gov/genomes/Apis_mellifera) were approximately 68.41% and 42.81% in *Apis mellifera* and *Apis cerana* samples, respectively (Table 1). In each library, those tags with a copy number greater than 100 occupy more than 80% of the clean tags, showing a narrow distribution of distinct clean tags. In contrast, those tags with a copy number between 2 and 5 showed a broad distribution (exceeding 50%) of distinct clean tags (Additional file 3: Figure S3).

**Table 1:** **Statistics of DGE sequencing at the three developmental stages**

Summary		NEW 1	NEW 2	Nurse 1	Nurse 2	Forager 1	Forager 2	Total
Raw tag	*Aml*	6,000,000	6,000,000	6,000,000	6,000,000	9,020,722	9,018,194	42,038,916
	*Acc*	6,000,000	6,000,000	6,000,000	6,000,000	6,000,000	6,000,000	36,000,000
Distinct tag	*Aml*	211,272	198,230	157,958	160,974	289,909	256,732	1,275,075
	*Acc*	215,863	219,554	144,442	130,883	170,217	213,917	1,094,876
Clean tag	*Aml*	5,882,059(98.03%)	5,890,202(98.17%)	5,910,901(98.52%)	5,911,105(98.52%)	5,423,816(60.13%)	5,849,152(64.86%)	34867235(82.94%)
	*Acc*	5,883,978(98.07%)	5,878,500(97.98%)	5,917,830(98.63%)	5,923,277(98.72%)	5,904,132(98.40%)	5,871,384(97.86%)	35,379,101(98.28%)
Distinct clean tag	*Aml*	100,676(47.65%)	95,104(47.98%)	77,435(49.02%)	80,286(49.88%)	61,548(21.23%)	52,987(20.64%)	468,036(36.71%)
	*Acc*	106,580(49.37%)	104,133(47.43%)	71,482(49.49%)	63,646(48.63%)	82,699(48.58%)	95,883(44.82%)	524,423(47.90%)
All tag mapping to gene	*Aml*	3,958,194(67.29%)	4,039,673(68.58%)	4,749,009(80.34%)	4,687,158(79.29%)	4,578,511(84.41%)	5,206,584(89.01%)	27,219,129(78.07%)
	*Acc*	2,476,714(42.09%)	2,570,588(43.73%)	2,740,538(46.31%)	2,755,741(46.52%)	2,422,568(41.03%)	3,091,826(52.66%)	16,057,975(45.39%)
Unambiguous tag mapping to gene	*Aml*	3,549,912(60.35%)	3,579,074(60.76%)	3,550,900(60.07%)	3,551,385(60.08%)	4,465,416(82.33%)	5,157,312(88.17%)	23,853,999(68.41%)
	*Acc*	2,370,309(40.28%)	2,512,797(42.75%)	2,520,841(42.60%)	2,321,965(39.20%)	2,404,037(40.72%)	3,014,975(51.35%)	15,144,924(42.81%)
Mapping to genome	*Aml*	1,100,732(18.71%)	1,025,724(17.41%)	732,560(12.39%)	751,100(12.71%)	558,499(10.30%)	458,366(7.84%)	4,626,981(13.27%)
	*Acc*	925,681(15.73%)	1,002,791(17.06%)	659,768(11.15%)	492,653(8.32%)	923,103(15.63%)	773,385(13.17%)	4,777,381(13.50%)
All tag-mapped genes	*Aml*	8,060(72.88%)	7,705(69.67%)	7,725(69.85%)	7,464(67.49%)	7,388(66.8%)	6,761(61.13%)	45,103(67.97%)
	*Acc*	7,317(66.16%)	7,385(66.77%)	6,656(60.18%)	6,450(58.32%)	6,961(62.94%)	7,045(63.7%)	41,814(63.01%)
Unambiguous tag-mapped genes	*Aml*	7,826(70.76%)	7,491(67.73%)	7,503(67.84%)	7,255(65.6%)	7,184(64.95%)	6,578(59.48%)	43,837(66.06%)
	*Acc*	7,077(63.99%)	7,157(64.71%)	6,469(58.49%)	6,250(56.51%)	6,743(60.97%)	6,824(61.7%)	40,520(61.06%)
Unknown tag	*Aml*	823,133(13.99%)	824,805(14.00%)	429,332(7.26%)	472,847(8.00%)	286,806(5.29%)	184,202(3.15%)	3,021,125(8.66%)
	*Acc*	2,481,583(42.18%)	2,305,121(39.21%)	2,517,524(42.54%)	2,674,883(45.16%)	2,558,461(43.33%)	2,006,173(34.17%)	14,543,745(41.11%)

To determine whether the depth of deep sequencing is sufficient, we performed a sequencing saturation analysis (Additional file 4: Figure S4). When the sequencing amount of the twelve DGE libraries reached a value close to 2 M, the number of detected genes reached a value near the limit, suggesting saturation of the sequencing depth.

#### DEGs between different developmental stages of the hypopharyngeal gland in Apis mellifera

**Figure 1:**
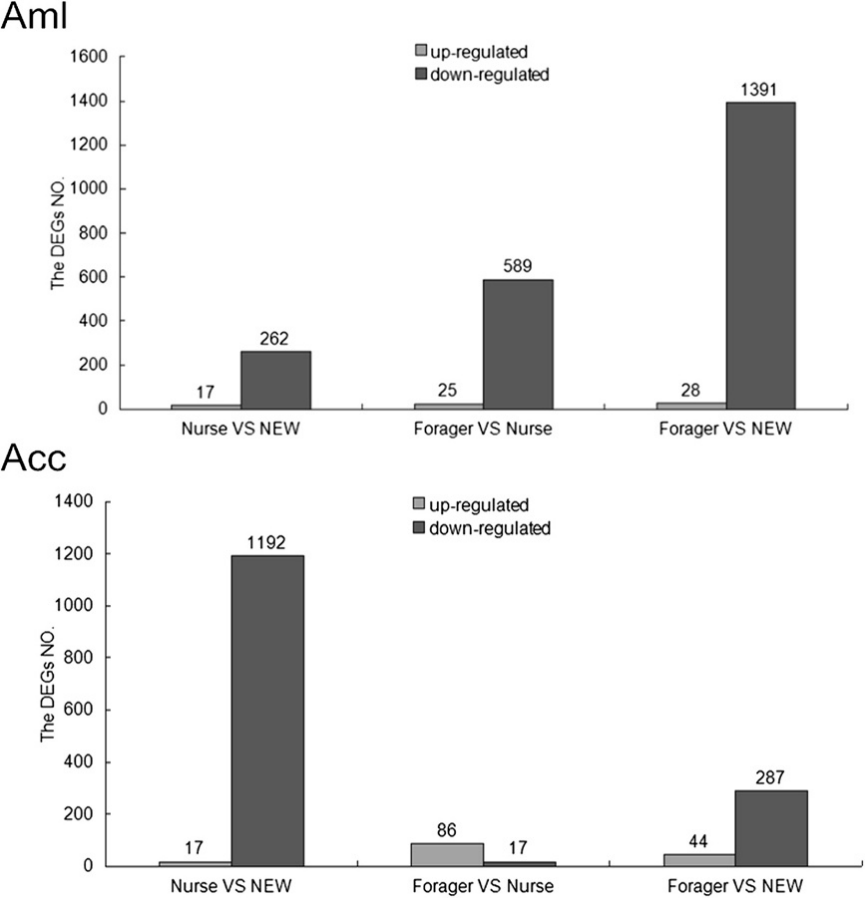
**DEGs between different developmental stages of HGs in** ***Apis mellifera*** **and** ***Apis cerana*** **.** NEW represents newly emerged worker, as in the other figures and tables.

At the three developmental stages of *Apis mellifera*, 8237 of the annotated genes were detected (Additional file 5: Figure S5). We then analyzed the gene expression differences between any two developmental stages of *Apis mellifera*. A total of 1482 genes showed an expression difference in at least one pairwise comparison. Of these, 279, 614, and 1419 genes were differentially expressed in the comparisons among newly emerged worker vs. nurse, nurse vs. forager, and newly emerged worker vs. forager (Figure 1, Additional file 6: Table S1), respectively. Among the three stages, 239, 9, and 17 genes showed their highest expression at the newly emerged worker, nurse and forager stages, respectively.

**Figure 2:**
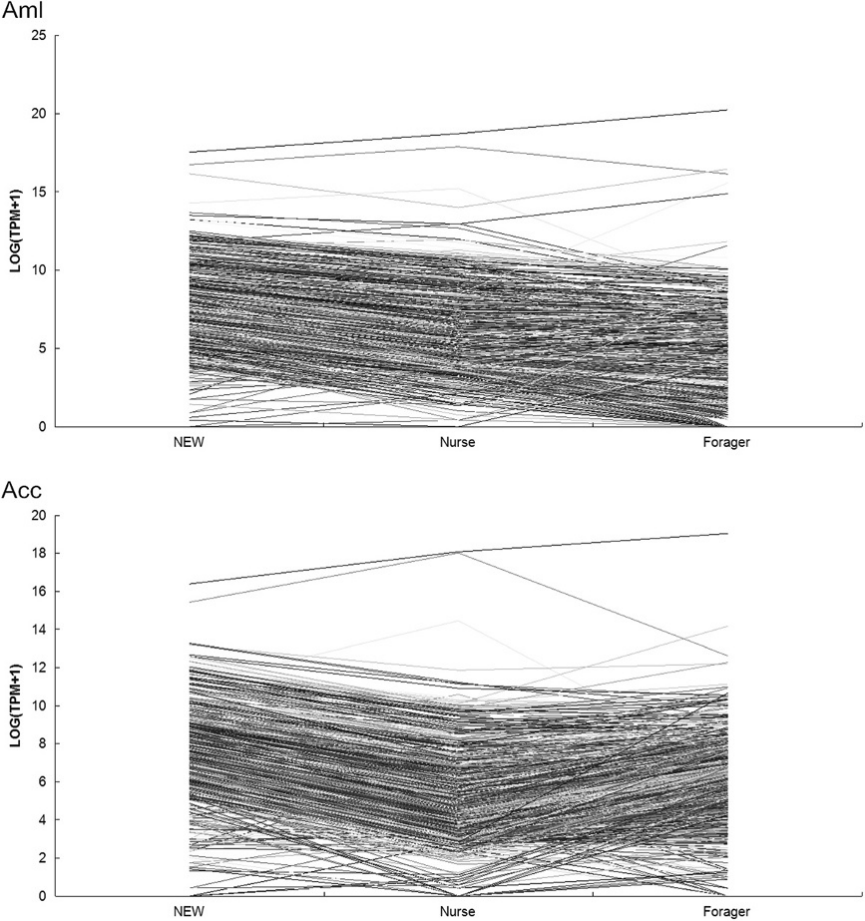
**Expression profile of the DEGs of HGs at the three developmental stages of** ***Apis mellifera*** **and** ***Apis cerana*** **.**

We analyzed the expression pattern of the 1482 DEGs at the three developmental stages of *Apis mellifera*. Contrary to our expectation, we haven’t observed a large number of up-regulated genes at the nurse stage. In general, the expression levels of the 1482 genes in *Apis mellifera* showed higher expression in the newly emerged worker bees and then gradually decreased with developmental progress (Figure 2).

We compared our results with those from previous proteomics studies performed by Feng *et al.* [8], in which 27 proteins were identified as differentially expressed between day 1 to day 20 in *Apis mellifera* HGs. Of these, 12 showed some expression difference in our study among the three developmental stages of HGs, and most of them showed a similar expression pattern to that reported by Feng *et al*. This result to some extent confirmed the reliability of our experimental results.

Because the transition from newly emerged worker bees to nurse bees is the critical period for royal jelly production, we paid more attention to the DEGs between these two stages. We found the alpha-amylase (NM_001011598.1) and alpha-glucosidase (NM_001011608.1), which have been repeatedly reported to be expressed specifically in the HGs of foragers and have been speculated to be related to the processing of nectar into honey [2, 3, 6], were significantly up-regulated in nurses compared with the newly emerged workers and continued to be up-regulated in foragers, which is consistent with the proteomics results reported by Li *et al.* [8]. These two enzymes are involved in the digestion of carbohydrates. Alpha-amylase is though to be needed to hydrolyze starch into glucose [15]. Alpha-glucosidase can catalyze polysaccharide digestion and function in the final steps of starch digestion [16]. The up-regulation of these two genes at the nurse stage may be related to some other function with the exception of brewing honey.

Although MRJPs are the major protein components of the royal jelly, we only found one MRJP member, namely MRJP7 (NM_001014429.1) expressed at its highest level at the nurse stage among the three stages. Moreover, MRJP1 (NM_001011579.1) and MRJP4 (NM_001011610.1) exhibited strong expression in the newly emerged workers. However, these two genes showed no statistically significant difference between the newly emerged workers and nurses, although their TPM values in nurses were higher than those found in the newly emerged workers. Feng *et al.* also found that MRJP1, 2 and 3 could be detected in the HGs of workers on day 1 [8]. These results suggested that the HGs of workers already have secretory activity before the nurse stage.

The GO enrichment analysis of the DEGs between newly emerged workers and nurses showed that 21 items were significantly enriched (P < 0.05) (Additional file 7: Table S2). The KEGG pathway enrichment analysis of the DEGs between these two stages indicated that “Ribosome”, “Protein processing in endoplasmic reticulum”, and “Protein export” (Additional file 8: Table S3), which are related to protein synthesis or secretion, were significantly enriched items (Qvalue < 0.05).

In the comparison between nurses and foragers, most of the DEGs are down-regulated in foragers. The GO enrichment analysis revealed that eight items, including “macromolecular complex”, “ribonucleoprotein complex”, “intracellular”, “intracellular part”, “structural molecule activity”, “metal cluster binding”, “metabolic process”, and “gene expression”, were significantly enriched (P < 0.05) (Additional file 7: Table S2). The KEGG pathway enrichment analysis revealed that “Ribosome”, “Metabolic pathways”, “Oxidative phosphorylation”, “Parkinson’s disease”, “Fatty acid metabolism”, and “Protein processing in endoplasmic reticulum” were significantly enriched items (Qvalue < 0.05) (Additional file 8: Table S3).

#### DEGs between different developmental stages of the hypopharyngeal gland in Apis cerana

In *Apis cerana*, 7486 genes were detected to be transcribed at the three stages (Additional file 5: Figure S5). A total of 1313 genes showed an expression difference at least between two stages. Of them, 1209, 103, and 331 genes showed an expression difference in the comparisons of newly emerged worker vs. nurse, nurse vs. forager and newly emerged worker vs. forager (Figure 1, Additional file 9: Table S4), respectively. A total of 254, 4, and 32 genes showed their highest expression at the newly emerged worker, nurse, and forager stages, respectively.

Similar to the findings found in *Apis mellifera*, the 1313 DEGs overall showed a higher expression in the newly emerged workers and decreased expression at the nurse stage. However, unlike the findings found in *Apis mellifera*, most of these DEGs were slightly up-regulated at the forager stage compared with the nurse stage (Figure 2). The expression pattern of the 1313 DEGs in *Apis cerana* and the 1482 DEGs in *Apis mellifera* indicated that even though the HGs exhibit their highest activity for royal jelly secretion at the nurse stage, but no peak of a large amount of up-regulated genes was appeared in nurse. This expression pattern is in fact in accordance with the physiological activity of the HGs and can be reasonably explained. At the newly emerged worker stage, the HGs are in a phase of rapid growth, and a large number of genes are expressed at a higher level to promote their development. At the nurse stage, however, although the size and secretory activity of the HGs reach their maximum, the resources of the HG cells are mainly used for the synthesis of royal jelly; therefore, those genes related to royal jelly protein synthesis and secretion are highly expressed, whereas the expression level of the other genes are decreased. At the forager stage, the HGs of honeybees begin to shrink and their secretion activity is decreased, which leads to the expression level of most of the genes in HGs remaining at a relatively lower level or exhibiting a further declined.

Of the DEGs found between newly emerged workers and nurses of *Apis cerana*, we found several major royal jelly protein genes, including MRJP1 (NM_001011579.1), MRJP5 (NM_001011599.1), MRJP6 (NM_001011622.1), and MRJP7 (NM_001014429.1), were significantly up-regulated at the nurse stage, which is consistent with their function in the HG. Alpha-glucosidase (NM_001011608.1) and alpha-amylase (NM_001011598.1) were also up-regulated in nurses.

The GO enrichment analysis of the DEGs between newly emerged workers and nurses showed that 53 items were significantly enriched (P < 0.05) (Additional file 10: Table S5). The KEGG pathway enrichment analysis indicated that 19 items, including the above-mentioned three items found between newly emerged workers and nurses in *Apis mellifera*, were significantly enriched (Qvalue < 0.05) (Additional file 11: Table S6). These results are consistent with the physiological changes of honeybee HGs during this period.

Between the nurse and forager stages, however, only one GO item namely “protein tyrosine/serine/threonine phosphatase activity”, was significantly enriched (Qvalue < 0.05) (Additional file 10: Table S5), and no KEGG items were significantly enriched (Qvalue < 0.05).

#### Gene expression difference in the hypopharyngeal gland between Apis mellifera and Apis cerana

**Figure 3:**
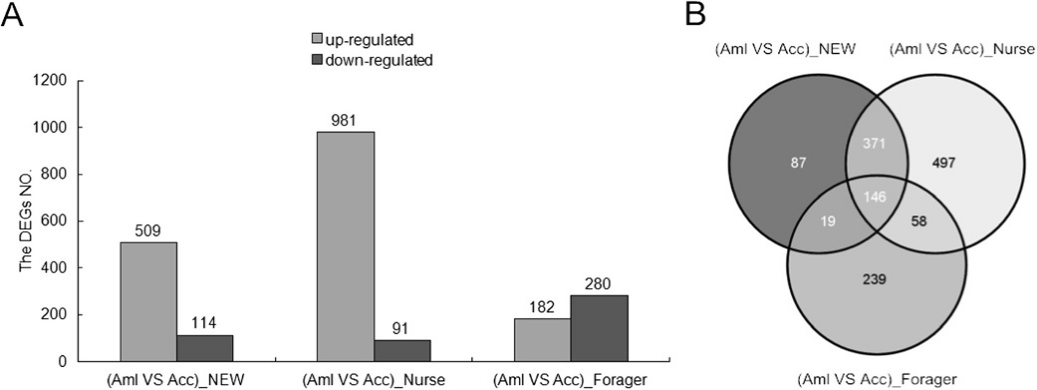
**DEGs between** ***Apis mellifera*** **and** ***Apis cerana*** **. (A)** Histogram of DEGs between *Apis mellifera* and *Apis cerana* at each developmental stage of HGs. **(B)** Venn diagram of DEGs between *Apis mellifera* and *Apis cerana* at each developmental stage of HGs.

**Figure 4:**
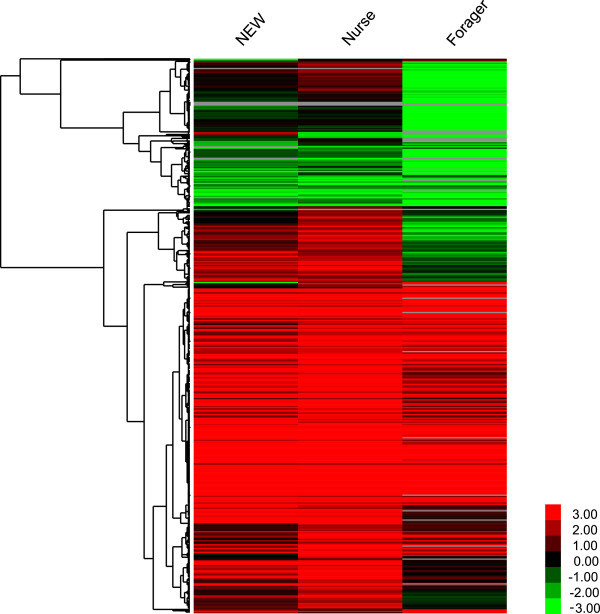
**Hierarchical clustering analysis of the 1417 DEGs between** ***Apis mellifera*** **and** ***Apis cerana*** **.** For each gene, the TPM mean value of the two biological replicates at each stage was used to calculate the expression ratio between *Apis mellifera* and *Apis cerana*, i.e., TPM _*Apis mellifera*_ */* TPM _*Apis cerana*._ If the value of TPM _*Apis cerana*_ was 0, it was replaced by 0.01. This ratio was log2-transformed and used for the clustering analysis, which was performed using the Cluster 3.0 and Treeview programs. Red represents up-regulated expression in *Apis mellifera*. Green represents up-regulated expression in *Apis cerana*.

We compared the gene expression difference in HGs between *Apis mellifera* and *Apis cerana* and identified 1417 DEGs between them (Figure 3, Additional file 12: Table S7). Of them, 623, 1072, and 462 genes showed an expression difference at the newly emerged, nurse, and forager stages, respectively. At the forager stage, more DEGs were up-regulated in *Apis cerana* compared to *Apis mellifera*, whereas at the newly emerged worker and nurse stages, the up-regulated DEGs in *Apis mellifera* were markedly higher than those found in *Apis cerana* (Figures 3 and 4). In particular, the nurse stage showed the highest number of differentially expressed genes between these two species, which could perhaps explain the phenomenon that the production of royal jelly in *Apis mellifera* is much higher than that observed in *Apis cerana*. Many of the 1417 DEGs exhibit important biological significance, including MRJPs and genes related to cell development and differentiation, such as IGF pathway genes and TOR pathway genes.

The GO enrichment analysis of all of the 1417 DEGs showed that “cytoplasmic part”, “cytoplasm”, “macromolecular complex”, “ribonucleoprotein complex”, “mitochondrion”, “mitochondrial part”, “structural molecule activity”, “metabolic process”, and “organic substance metabolic process” are dominant (P < 0.05) (Additional file 13: Table S8). The KEGG pathway enrichment analysis indicated that “Ribosome”, “Metabolic pathways”, “Oxidative phosphorylation”, “Parkinson’s disease”, “Fatty acid metabolism”, “Valine, leucine and isoleucine degradation”, and “Protein processing in endoplasmic reticulum” were significantly enriched (Qvalue < 0.05) (Additional file 14: Table S9).

### MRJPs

**Figure 5:**
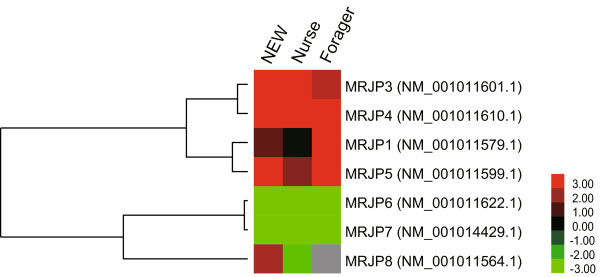
**Hierarchical clustering analysis of the differentially expressed MRJPs between** ***Apis mellifera*** **and** ***Apis cerana*** **.** For each gene, the TPM mean value of the two biological replicates at each stage was used to calculate the expression ratio between *Apis mellifera* and *Apis cerana*, i.e., TPM _*Apis mellifera*_ */* TPM _*Apis cerana*._ If the value of TPM _*Apis cerana*_ was 0, it was replaced by 0.01. This ratio was log2-transformed and used for the clustering analysis, which was performed using the Cluster 3.0 and Treeview programs. Red represents up-regulated expression in *Apis mellifera*. Green represents up-regulated expression in *Apis cerana*.

The MRJPs are the main protein components of royal jelly. Nine MRJP-encoding genes (MRJP1-9) have been identified from the honeybee genome. Our results showed that most members of the MRJPs (i.e., MRJP1-9 with the exception of MRJP2 and MRJP9) showed an expression difference between *Apis mellifera* and *Apis cerana* (Figure 5). Of these, the MRJP1 (NM_001011579.1) showed a significantly higher expression level in *Apis mellifera* than in *Apis cerana* at the newly emerged worker and forager stages but showed no significant expression difference at the nurse stage. MRJP1 is the most abundant protein in royal jelly (occupying 31%) and the key factor for the induction of queen and worker differentiation [17]. We can speculate that the HGs of nurse bees of both *Apis mellifera* and *Apis cerana* need to synthesize a large amount of MRJP1 to maintain a basic function of the royal jelly. In addition to MRJP1, MRJP3 (NM_001011601.1) was also found to be expressed at a higher level in *Apis mellifera* at the newly emerged worker and nurse stages. MRJP4 (NM_001011610.1) and 5 (NM_001011599.1) were constantly expressed at higher levels in *Apis mellifera* at all three stages. In contrast, MRJP6 (NM_001011622.1), MRJP7 (NM_001014429.1), and MRJP8 (NM_001011564.1) exhibited the opposite trend; MRJP6 and MRJP7 were constantly expressed at higher levels in *Apis cerana* at all of the stages, and MRJP8 was expressed at a higher level at the forager stage. MRJP1-5 account for up to 90% of the most abundant proteins of royal jelly and have been repeatedly suggested to mainly have a nutritional function [18–20]. Thus, the high expression levels of MRJP3, 4, and 5 in *Apis mellifera* are consistent with their nutritional function. Nevertheless, the high expression of MRJP6, 7 and 8 in *Apis cerana* may be due to the non-nutritional function of MRJPs, as has been reported in many studies [21–27]. For example, a previous expression analysis reported that MRJP1, 2 and 7 can be detected in mushroom bodies [21–23]. MRJP1 and 3 are also expressed in drones (head, body, and larvae) and queens (ovary and larvae) [24]. More surprisingly, MRJP8 and 9, which are rare in RJ, could be detected in honeybee venom [25–27]. All of the expression data lead to the conclusion that MRJPs have important functions in general honeybee physiology in addition to just their nutritional value for developing larvae.

### Ribosomal proteins

**Figure 6:**
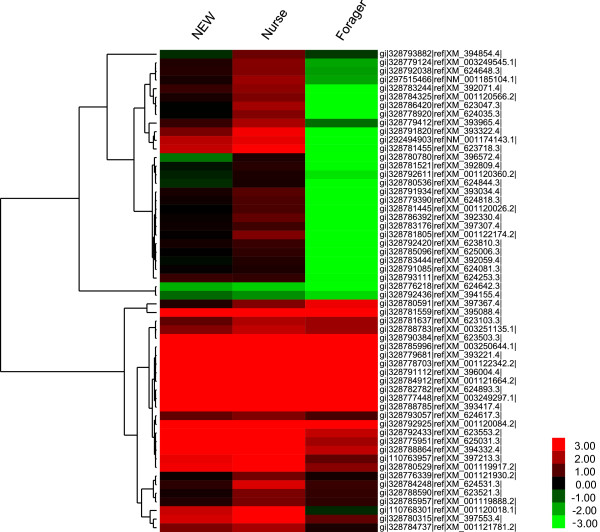
**Hierarchical clustering analysis of the 56 differentially expressed ribosomal protein genes between** ***Apis mellifera*** **and** ***Apis cerana*** **.** For each gene, the TPM mean value of the two biological replicates at each stage was used to calculate the expression ratio between *Apis mellifera* and *Apis* cerana, i.e., TPM _*Apis mellifera*_ */* TPM _*Apis cerana*._ If the value of TPM _*Apis cerana*_ was 0, it was replaced by 0.01. This ratio was log2-transformed and used for clustering analysis, which was performed using the Cluster 3.0 and Treeview programs. Red represents up-regulated expression in *Apis mellifera*. Green represents up-regulated expression in *Apis cerana*.

Ribosomal proteins form the two subunits of the ribosome together with the rRNAs and play an important role in intracellular protein synthesis [28]. Of the DEGs, a total of 56 ribosomal protein genes, nearly one-third of all of the ribosomal protein genes in the honeybee genome, showed differential expression between *Apis mellifera* and *Apis cerana*. Of them, 23, 40, and 26 showed an expression difference at the newly emerged worker, nurse, and forager stages, respectively. The gene expression cluster analysis indicated that most of these ribosomal protein genes were up-regulated in *Apis mellifera* at the newly emerged worker and nurse stages (Figure 6). In particular at the nurse stage, the fold expression difference found for most of these ribosomal protein genes reached a maximum among the three stages. This finding implies that protein synthesis in the HGs of *Apis mellifera* is more vigorous than that in *Apis cerana*.

### TOR, insulin/IGF and TGF pathway genes

Because the size of the *Apis mellifera* HG is larger than that of *Apis cerana* [12], we speculated that some genes related to cell growth and differentiation may contribute to this difference; thus, more attention was paid to genes in related signaling pathways, such as the TOR, insulin/IGF and TGF pathways. Among the 1417 DEGs, we found that two TOR pathway genes, namely 3-phosphoinositide-dependent protein kinase 1 (PDK1) (XM_394208.4) and eukaryotic translation initiation factor 4E (XM_624287.3), and two insulin/IGF pathways genes, namely IGF-II mRNA-binding protein (XM_393878.4) and cell growth-regulating nucleolar protein-like (XM_623800.3), were significantly expressed higher in *Apis mellifera* than in *Apis cerana* at the newly emerged worker and nurse stages (Additional file 12: Table S7). The TOR and insulin/IGF signaling pathways have been identified as two main pathways that control cell growth through studies in model organisms [29]. The TOR pathway acts as a nutrient sensor in multicellular organisms and regulates growth in response to nutrients, and the insulin/IGF pathway is involved in coordinating cellular growth in response to endocrine signals and plays a key role in regulating growth in invertebrates and vertebrates [29]. The insulin and TOR pathways form a signaling network that integrates information about nutrient availability and an intrinsic developmental program. In addition, the TGF-beta receptor 1 genes (XM_003251608.1) were also expressed at higher levels in *Apis mellifera* at the newly emerged worker and nurse stages. The TGF-β signaling pathway has been implicated as an important regulator of almost all major cell behaviors, including proliferation, differentiation, cell death, and motility [30]. The higher expression of these genes in *Apis mellifera* may suggest that the up-regulation of these genes can promote the development of HGs, which to some extent leads to the higher yield of royal jelly.

## Conclusions

This study provides the first report of some DEGs in the hypopharyngeal gland between *Apis mellifera* and *Apis cerana* at the newly emerged worker, nurse and forager stages. Our results confirmed that many DEGs may play an important role in the development of HGs and the secretion of royal jelly. All of the information obtained in our study contributes to further research on the specifically expressed genes in HG at the molecular level.

## Methods

### Insect

The honeybee species *Apis mellifera ligustica* and *Apis cerana cerana* were used in this experiment. They were bred in the Honeybee Research Institute, Jiangxi Agricultural University, China (28.46 °N, 115.49 °E). Worker bees from these two species were gathered at the three developmental stages (newly emerged worker, nurse and forager). The foragers could be easily identified by the pollen loads on their hind legs. The nurses were caught at the time when they entered the cells and were nursing the larvae. For each developmental stage, two independent biological replicates were collected. Finally, a total of 720 workers were sampled randomly. All of the samples were collected alive, immediately flash frozen in liquid nitrogen, and then stored at −80°C until further processing. The HGs were dissected under a binocular stereo microscope. The detailed dissection steps are as follows: First the labrum was gripped with curved forceps to fix the head, and the skull of the head was then exscinded with a razor blade. After removing the shell on the cranial cavity using forceps, we instilled a few drops of normal saline (137 mmol/L NaCl, 2.7 mmol/L KCl, 10 mmol/L Na_2_HPO_4_, and 2 mmol/L KH_2_PO_4_) to ensure that the HGs dissociate from the brain tissue and then selected the HGs and cut them off from the mouthpiece. Finally, the HGs were rinsed with DEPC-treated water and promptly frozen with liquid nitrogen for Illumina sequencing analysis of the DGE. During dissection, the room temperature was maintained at 16°C, and the normal saline and DEPC-treated water were kept on ice. To prevent the degradation of mRNA, the sampled honeybee heads were preserved in dry ice before dissection and the HGs were dissected out within 4 min. The HGs from 60 worker bees were pooled as a sample to create the tag library.

### Digital gene expression library preparation and sequencing

The total RNA was extracted using the SV Total RNA isolation System (Promega, USA) and then subjected to quality inspection. The tag-seq libraries were then constructed using the Illumina Gene Expression Sample Prep Kit according to the manufacturer’s instruction. Briefly, mRNA was purified from 6 μg of total RNA with oligo (dT) magnetic beads and then synthesized into double-stranded DNA (cDNA) by reverse transcription. The cDNA was digested with *Nla* III which could recognize CATG site and the Illumina adaptor 1 was ligated to the sticky 5′ end of the digested bead-bound 3′ cDNA fragments. The junction of Illumina adaptor 1 and the CATG site is the recognition site of *Mme* I, which is a type of endonuclease with separated recognition sites and digestion sites and cuts 17 bp downstream of the CATG site, producing tags containing adaptor 1. Then, Illumina adaptor 2 was ligated to the 3′ ends of the tags, obtaining tags with different adaptors on both ends. The cDNA tags containing adaptors 1 and 2 were enriched with 15-cycle PCR amplification with the sequencing primers and then purified by 6% PAGE gel electrophoresis. The single-stranded molecules were bound to the Illumina sequencing chip and sequenced using Illumina HiSeq™ 2000. The sequencing-received raw image data were transformed by base calling into sequence data and stored in FASTQ format. Each tunnel generated millions of raw reads with a sequencing length of 49 bp. The raw sequences were filtered into clean tags by the process, which included the removal of the adaptor sequence, empty tags, low-quality tags, tags with only one copy number and tags that were too long or too short, leaving tags 21 bp in length, which were named clean tags. The clean reads of *Apis mellifera* and *Apis cerana* were submitted to the NCBI Sequence Read Archive database under the accession numbers SRP033111 and SRP033303, respectively.

### Tag mapping and statistical analysis

Before mapping tag to reference sequences, two tag libraries containing all of the possible CATG + 17 nt tag sequences were created as reference tag databases using all of the available mRNA sequences and genome sequences of *A. mellifera* downloaded from the GenBank database (ftp://ftp.ncbi.nih.gov/genomes/Apis_mellifera). Because *A. mellifera* and *A. cerana* are two closely related species, we used mRNA and genome sequences of *A. mellifera* as the reference sequences of *A. cerana*. All of the clean tags were then mapped to the reference tag database with only one nucleotide mismatch being allowed, and unambiguous tags were annotated. The number of unambiguous clean tags for each gene was calculated and normalized to TPM (number of transcripts per million clean tags). Those tags that cannot be mapped to any gene in the tag database of mRNA sequences were continuing mapped to the tag database of the reference genome sequence.

### Identification of differentially expressed genes

To identify the differentially expressed genes (DEGs) among the sample libraries, we applied a rigorous statistical algorithm based on the protocol from Tarazona and García-Alcalde [31]. The NOISeq method for the analysis of the “noise” distribution from the actual data could be better adapted to the size of the dataset and more effective for controlling the false discovery rate (FDR). Briefly, let X^g^_ij_ be the expression of gene *g* in condition *i* (*i* = 1, 2) and replicate *j*. The log fold change  and the difference  are used to measure the expression level change between the two conditions. To determine the probability of differential expression, the algorithm creates a so-called “noise” distribution by pooling all of the replicates’ the empirical cumulative distribution function F (M_n_, D_n_) values within the same condition. The random variables describing the noise distribution can be regarded as F (|M*|, |D*|). A gene *g* is considered to be differentially expressed when the corresponding values for |M^g^| and |D^g^| are likely to be higher than that due to noise (|M*|and D* values). The probability can be written as P_1_ (|M*| < |M^g^|, |D*| < |D^g^|) and the probability of not being differentially expressed between the two conditions can be easily derived as P_0_ = 1-P_1_. The higher this probability, the higher the expression changes between conditions. We used a probability threshold of Q = 0.8, which is equivalent to an odds of 4:1 (P_1_/P_0_), which means that the gene is four-fold more likely to be differentially expressed than non-differentially expressed. The genes with a Q value ≥ 0.8 and an absolute value of log2 Ratio ≥ 1 were considered to be significantly expressed genes.

Finally, the identified DEGs were used for GO and KEGG pathway analysis. The GO enrichment analysis of functional significance was conducted using a hypergeometric test that mapped all of the DEGs to terms in the GO database (http://geneontology.org). The formula used for the calculation is the following:

where *N* is the number of all genes with a GO annotation, *n* is the number of differentially expressed genes in *N. M* is the number of all genes that are annotated to the certain GO terms, and *m* is the number of differentially expressed genes in *M*.

The KEGG pathway enrichment analysis identified significantly enriched metabolic pathways or signal transduction pathways in the DEGs compared with the whole-genome background. The formula used is the same as that used in GO analysis.

The cluster analysis of gene expression patterns was performed using the cluster 3.0 software and the “Java Treeview” software. For each gene, the TPM mean value of the two biologically replicates at each stage were used for cluster analysis.

## Electronic supplementary material

Additional file 1: Figure S1: Reproducibility of the expression level between replicates. The TPM value of replicate 1 is plotted on the x-axis, and the TPM value of replicate 2 is plotted on the y-axis. (EPS 4 MB)

Additional file 2: Figure S2: Distribution of total tags and distinct tags over different tag abundance categories in each sample. The numbers and percentage of tags containing N, tags with adaptor only, tags with copy number < 2 and clean tags are shown. (EPS 12 MB)

Additional file 3: Figure S3: Distribution of total clean tags and distinct clean tags over different tag abundance categories in each sample. The numbers in the square brackets indicate the range of copy numbers for a specific category of tags. For example [2, 5], means that all of the tags in this category have 2 to 5 copies. The numbers in the parentheses of the left and right graphs show the total copy number of the clean tags and the total types of clean tags respectively in that category. (EPS 15 MB)

Additional file 4: Figure S4: Saturation analysis of the clean tags. (EPS 6 MB)

Additional file 5: Figure S5: Distribution of developmental stage-specific and co-expressed annotated genes in *Apis mellifera* and *Apis cerana*. (EPS 3 MB)

Additional file 6: Table S1: DEGs between HGs of *Apis mellifera* at different developmental stages. (XLS 1 MB)

Additional file 7: Table S2: Gene ontology enrichment analysis of the 1482 DEGs in *Apis mellifera.* (XLS 160 KB)

Additional file 8: Table S3: KEGG pathway enrichment analysis of the 1482 DEGs in *Apis mellifera*. (XLS 68 KB)

Additional file 9: Table S4: DEGs between HGs of *Apis cerana* at different developmental stages. (XLS 938 KB)

Additional file 10: Table S5: Gene ontology enrichment analysis of the 1313 DEGs in *Apis cerana*. (XLS 327 KB)

Additional file 11: Table S6: KEGG pathway enrichment analysis of the 1313 DEGs in *Apis cerana*. (XLS 68 KB)

Additional file 12: Table S7: The 1417 DEGs between *Apis mellifera* and *Apis cerana*. (XLS 1 MB)

Additional file 13: Table S8: Gene ontology enrichment analysis of the 1417 DEGs between *Apis mellifera* and *Apis cerana*. The results were summarized in three main categories: biological process, cellular component and molecular function. (XLS 40 KB)

Additional file 14: Table S9: KEGG pathway enrichment analysis of the 1417 DEGs between *Apis mellifera* and *Apis cerana*. (XLS 32 KB)
